# Pre‐ and post‐treatment blood‐based genomic landscape of patients with *ROS1* or *NTRK* fusion‐positive solid tumours treated with entrectinib

**DOI:** 10.1002/1878-0261.13214

**Published:** 2022-04-22

**Authors:** Rafal Dziadziuszko, Tiffany Hung, Kun Wang, Voleak Choeurng, Alexander Drilon, Robert C. Doebele, Fabrice Barlesi, Charlie Wu, Lucas Dennis, Joel Skoletsky, Ryan Woodhouse, Meijuan Li, Ching‐Wei Chang, Brian Simmons, Todd Riehl, Timothy R. Wilson

**Affiliations:** ^1^ Department of Oncology and Radiotherapy Medical University of Gdańsk Gdańsk Poland; ^2^ Oncology Biomarker Department Genentech, Inc. South San Francisco CA USA; ^3^ Biostatistics Foundation Medicine Inc. Cambridge MA USA; ^4^ Oncology Biostatistics Genentech, Inc. South San Francisco CA USA; ^5^ Early Drug Development Service Memorial Sloan Kettering Cancer Center, and Weill Cornell Medical College New York NY USA; ^6^ Anschutz Medical Campus University of Colorado Aurora CO USA; ^7^ The National Centre for Scientific Research (CNRS) The National Institute of Health and Medical Research (INSERM) Aix Marseille University Marseille France; ^8^ 46657 Medical Oncology Gustave Roussy Villejuif France; ^9^ Franchise Development Foundation Medicine Inc. Cambridge MA USA; ^10^ Companion Diagnostics Development Foundation Medicine Inc. Cambridge MA USA; ^11^ Regulatory Affairs Foundation Medicine Inc. Cambridge MA USA; ^12^ Biometrics and Biomarkers Foundation Medicine Inc. Cambridge MA USA; ^13^ Product Development Oncology Genentech, Inc. South San Francisco CA USA

**Keywords:** F1L CDx, genomic profiling, *NTRK*, resistance, *ROS1*

## Abstract

Genomic tumour profiling informs targeted treatment options. Entrectinib is a tyrosine kinase inhibitor with efficacy in *NTRK* fusion‐positive (‐fp) solid tumours and *ROS1*‐fp non‐small cell lung cancer. FoundationOne® Liquid CDx (F1L CDx), a non‐invasive *in* *vitro* next‐generation sequencing (NGS)‐based diagnostic, detects genomic alterations in plasma circulating tumour DNA (ctDNA). We evaluated the clinical validity of F1L CDx as an aid in identifying patients with *NTRK*‐fp or *ROS1*‐fp tumours and assessed the genomic landscape pre‐ and post‐entrectinib treatment. Among evaluable pre‐treatment clinical samples (*N* = 85), positive percentage agreements between F1L CDx and clinical trial assays (CTAs) were 47.4% (*NTRK* fusions) and 64.5% (*ROS1* fusions); positive predictive value was 100% for both. The objective response rate for CTA^+^ F1L CDx^+^ patients was 72.2% in both cohorts. The median duration of response significantly differed between F1L CDx^+^ and F1L CDx^−^ samples in *ROS1*‐fp (5.6 vs. 17.3 months) but not *NTRK*‐fp (9.2 vs. 12.9 months) patients. Fifteen acquired resistance mutations were detected. We conclude that F1L CDx is a clinically valid complement to tissue‐based testing to identify patients who may benefit from entrectinib and those with acquired resistance mutations associated with disease progression.

## Graphical abstract

Entrectinib is a kinase inhibitor that targets both the *NTRK* and *ROS1* oncogenes. We show that *NTRK* and *ROS1* fusions are present in 46% and 64%, respectively, of blood samples from patients enrolled on a clinical trial, with a response rate of 72%. Profiling blood at progression showed that resistance occurs via mutations in *NTRK*/*ROS1* or via downstream MAP‐kinase reactivation.

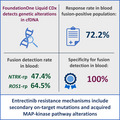

**Abbreviations**
cfDNA: circulating cell‐free DNA
CI: confidence interval
CNS: central nervous system
CR: complete response
CRC: colorectal cancer
CTA: clinical trial assay
ctDNA: circulating tumour DNA
DoR: duration of response
ECOG PS: Eastern Cooperative Oncology Group performance status
F1L CDx: FoundationOne® Liquid CDx
FDA: food and drug administration
fn: fusion‐negative
fp: fusion‐positive
IQR: interquartile range
LoD: limit of detection
MAPK: mitogen‐activated protein kinase
MASC: mammary analogue secretory carcinoma
N/A: not applicable
NE: non‐evaluable
NGS: next‐generation sequencing
NPA: negative per cent agreement
NPV: negative predictive value
NSCLC: non‐small cell lung cancer
NTRK: neurotrophic tyrosine receptor kinase
ORR: overall response rate
PD: progressive disease
PPA: positive per cent agreement
PPV: positive predictive value
PR: partial response
RECIST: response evaluation criteria in solid tumours
ROS1: ROS proto‐oncogene 1
SD: stable disease
TKI: tyrosine kinase inhibitor
TRK: tropomyosin receptor kinase

## 1 Introduction

The increasing use of targeted cancer treatments tailored to a tumour’s individual genomic profile has generated a need to develop rapid and reliable assays for comprehensive genomic profiling [1]. Next‐generation sequencing (NGS) is increasingly used to detect molecular alterations driving tumour development [2], and the genomic information provided can support physicians in making informed decisions to ensure patients have access to the most appropriate and effective treatment.

Tumour biopsies represent the most widely utilized source of tumour DNA for genomic analysis; while current tissue‐based assays show high sensitivity and specificity, several limitations are associated with solid tissue biopsies [3]. The invasiveness of biopsies makes it unsuitable for use in frail patients and repeat sampling for longitudinal tumour monitoring is undesirable. Solid tumour biopsies also cannot be taken from physically inaccessible tumours [3, 4], such as thoracic malignancies. Inherent tumour heterogeneity also means that solid tumour biopsies may fail to capture the complete genomic profile of an individual tumour [3, 5].

Technological advances have resulted in the development of liquid biopsy assays using circulating tumour DNA (ctDNA), a subset of circulating cell‐free DNA (cfDNA), extracted from blood plasma samples, as a basis for genomic analysis [6]. Liquid biopsy assays offer a minimally invasive means for genomic evaluation that is suitable for use in all patients for whom blood can be safely drawn, regardless of tumour location and patient frailty [7, 8]. Blood samples can also be taken easily, with lower burden on healthcare resources and greater patient convenience. As ctDNA is shed into blood plasma from each metastatic tumour site, a more complete picture of tumour heterogeneity is gained compared with tissue‐based sampling methods [7]. Because blood samples can be taken repeatedly for testing, liquid biopsy assays can support detection of actionable driver mutations to inform treatment selection at diagnosis, but can also monitor treatment response, detect recurrence, and identify resistance mechanisms, thereby potentially guiding therapeutic decisions [9, 10].

The FoundationOne® Liquid CDx (F1L CDx) assay (Foundation Medicine Inc., Cambridge, MA, USA) is an NGS‐based *in* *vitro* diagnostic for detecting genomic alterations in cfDNA, including gene fusions [11]. The F1L CDx assay received US Food and Drug Administration (FDA) approval as a comprehensive pan‐tumour liquid biopsy test for patients with solid tumours (August 2020) and has since received approval as a companion diagnostic in multiple indications, including non‐small cell lung cancer (NSCLC; alectinib, gefitinib, osimertinib, erlotinib), breast cancer (alpelisib), prostate cancer (olaparib, rucaparib) and ovarian cancer (rucaparib) [12].

Gene fusions of the neurotrophic tyrosine receptor kinase gene (*NTRK1/2/3*; coding for tropomyosin receptor kinases TRKA/B/C) and the tyrosine receptor kinase ROS proto‐oncogene 1 (*ROS1*) can constitutively activate kinases acting as oncogenic drivers [13, 14, 15, 16]. Entrectinib is a central nervous system (CNS)‐active potent inhibitor of TRKA/B/C and ROS1 [17, 18, 19] approved for the treatment of adult and paediatric patients aged ≥12 years with *NTRK* fusion‐positive (*NTRK*‐fp) solid tumours and adults with *ROS1* fusion‐positive (*ROS1*‐fp) NSCLC. This was based on an integrated analysis of three Phase I/II studies (ALKA‐372‐001, EudraCT 2012–000148–88; STARTRK‐1, NCT02097810; STARTRK‐2, NCT02568267), showing strong clinical efficacy with entrectinib in patients with *NTRK*‐fp solid tumours [objective response rate (ORR), 57%; median duration of response (DoR), 10.4 months] and *ROS1*‐fp NSCLC (ORR, 77%; median DoR 24.6 months) [17, 20].

This study examined the clinical validity of F1L CDx to aid in identifying patients with *NTRK*‐fp solid tumours and *ROS1*‐fp NSCLC for entrectinib treatment and assessed the pre‐ and post‐treatment genomic landscape of patients with *NTRK*‐fp solid tumours and *ROS1*‐fp NSCLC receiving entrectinib.

## 2 Materials and methods

### 2.1 Patient population

Patients in the analysis were enrolled in the Phase II global basket study STARTRK‐2 (NCT02568267). The design of this ongoing trial has been described previously [17, 20]. Briefly, patients aged ≥18 years with locally advanced/metastatic *NTRK*‐fp solid tumour (96% metastatic) or *ROS1*‐fp NSCLC (94% metastatic) measurable by Response Evaluation Criteria in Solid Tumors (RECIST; v1.1) had received no prior tyrosine kinase inhibitor (TKI) therapy, had an Eastern Cooperative Oncology Group performance status (ECOG PS) ≤ 2, and could be enrolled with brain metastases if they were asymptomatic or had received previous treatment resulting in symptom control. Patients received entrectinib 600 mg orally once daily, until documented radiographic progression, unacceptable toxicity or consent withdrawal. The high‐level tumour types included in the *NTRK*‐fp cohort are presented in Table S1. Primary endpoints were ORR and median DoR, assessed by blinded independent central review using RECIST v1.1. As part of the study scheduled assessments, blood samples were collected at screening, day 1 of each treatment cycle and at end of treatment.

The presence of *NTRK* or *ROS1* fusions was confirmed by tumour DNA‐ or RNA‐based clinical trial assays (CTAs) before enrolment in STARTRK‐2. The CTAs utilized comprised Trailblaze Pharos™ (Ignyta, F. Hoffmann‐La Roche Ltd, Basel, Switzerland) NGS central testing or local diagnostic laboratory nucleic acid‐based methodologies (e.g. NGS, Sanger sequencing, reverse transcription‐polymerase chain reaction, NanoString). If patients were enrolled via a local test and a tumour sample was available, independent central molecular NGS testing was performed post‐enrolment via the Trailblaze Pharos™ assay. For the *NTRK*‐fp cohort, one central and 18 local testing laboratories were used to enrol the study participants, using the following technologies: PCR (*n* = 1), NanoString (*n* = 1), RNA‐NGS (*n* = 29), DNA‐NGS (*n* = 19) and RNA‐ + DNA‐NGS (*n* = 4). For the *ROS1*‐fp cohort, one central and 11 local testing laboratories were used to enrol the study participants, using the following technologies: FISH (*n* = 15), RNA‐NGS (*n* = 27), DNA‐NGS (*n* = 9).

STARTRK‐2 was conducted in accordance with the principles of the Declaration of Helsinki and Good Clinical Practice guidelines. Written, informed consent was obtained from all patients. The study protocol and biosample collection were approved by all relevant institutional review boards and/or ethics committees.

### 2.2 Plasma samples

Patients with *NTRK*‐fp solid tumours or *ROS1*‐fp NSCLC enrolled in STARTRK‐2 (clinical cut‐off: May 2018) for whom frozen pre‐treatment plasma samples were available were eligible for inclusion in clinical bridging analyses. Those who also had available plasma samples at/after progressive disease (PD) on entrectinib treatment were eligible for resistance mutation analysis. Plasma was isolated from whole blood specimens at the time of collection and stored frozen until processing.

Additional paired plasma samples from patients with *NTRK* or *ROS1* fusion‐negative (fn) non‐NSCLC tumour types (*n* = 8) were purchased from an external commercial vendor (BiolVT LCC, Westbury, NY, USA).

### 2.3 Evaluation of plasma samples using the F1L CDx assay

The F1L CDx assay is a qualitative assay using targeted, high‐throughput hybridization‐based capture technology and is FDA‐approved to detect substitutions, insertions, and deletions in 311 genes, rearrangements in three genes, and copy number alterations in three genes [12]. cfDNA isolated from plasma is utilized and has a median limit of detection (LoD; % variant allele frequency) for rearrangements of 0.37% in enhanced and 0.90% in standard sensitivity regions. Reproducibility for detecting gene rearrangement is 99.2% [95% confidence interval (CI): 99.1–99.3] [11].

Analysis of all pre‐treatment and PD plasma samples using the F1L CDx assay was performed in the Foundation Medicine Inc. laboratory, according to standard workflow [11, 12]. Plasma was separated from whole blood by centrifugation and cfDNA subsequently isolated using the KingFisher™ Flex Magnetic Particle Processor (Thermo Fisher, Waltham, MA, USA) and built into genomic libraries. Libraries were sequenced using the Illumina NovaSeq 6000 platform (Illumina Inc., San Diego, CA, USA). Only samples with cfDNA content >30 ng, as assessed by the Agilent 4200 TapeStation assay, were included in the analyses.

### 2.4 Clinical bridging analyses

#### 2.4.1 Concordance analysis

Concordance between CTA and F1L CDx assays in detection of *NTRK* or *ROS1* fusions was calculated as positive and negative percent agreement (PPA; NPA). Positive and negative predictive values (PPV; NPV) were derived from PPA and NPA following adjustment for fusion prevalence (*NTRK*, 0.32% [21]; *ROS1*, 1–2% [15, 16]).

$$\mathrm{PPV}\;=\text{Pr}\left({\text{CTA}}^{+}\;¦\;{\text{F1L CDX}}^{+}\right)=\frac{\varphi *\mathrm{PPA}}{\left(\varphi *\mathrm{PPA}+\left(1‐\varphi \right)*\left(1‐\mathrm{NPA}\right)\right)}$$

$$\mathrm{NPV}\;=\text{Pr}\left({\text{CTA}}^{‐}\;¦\;{\text{F1L CDx}}^{‐}\right)=\frac{\left(1‐\varphi \right)*\mathrm{NPA}}{\left(\varphi *\left(1‐\mathrm{PPA}\right)+\left(1‐\varphi \right)*\mathrm{NPA}\right)}$$

Since the PPV point estimate is 1 and no statistical method is available, 18 was used as the *n* in the binomial distribution for a positive fusion number. The 95% CI was estimated by the Wilson score interval for PPV and the bootstrap method for NPV.

#### 2.4.2 Clinical efficacy analysis

Clinical efficacy (ORR, DoR) was calculated for the following populations: CTA^+^, CTA^+^ F1L CDx^+^, CTA^+^ F1L CDx^−^ and CTA^+^ F1L CDx unevaluable populations. Estimated clinical efficacy was then compared with entrectinib efficacy data from the primary integrated analysis of three Phase I/II studies enrolling patients with *NTRK*‐fp solid tumours or *ROS1‐*fp NSCLC [17, 20]. Two patients were removed from the original population due to prior crizotinib treatment and were therefore excluded from this analysis.

Let θ denote the estimate of overall response rate (ORR) for the FoundationOne Liquid CDx (F1L CDx)^+^ sub‐population. Given the concordance between CTAs and F1L CDx, θ was estimated using the following formula: [22]

θ=θ1∗P+θ2∗(1‐P)

where

- θ1 is the estimated ORR for (F1L CDx^+^ & CTA^+^)
- θ2 is the estimated ORR for (F1L CDx^+^ & CTA^−^)
- *P* is the conditional probability; *P =* Pr (CTA^+^│F1L CDx^+^)

As samples obtained from patients enrolled in STARTRK‐2 were enriched for CTA^+^ results, $\hat{P}$ and its corresponding variance var($\hat{P}$) were estimated by:

$$p=\frac{\varphi \omega_{11}}{\varphi \omega_{11}+\left(1‐\varphi \right)\omega_{10}}$$

where

- φ=prevalence of *NTRK* or *ROS1* in the intended use population
- $\omega_{11\;}=\mathrm{pr}\left(F1L\;{\mathrm{CDx}}^{+}\;\&\;{\mathrm{CTA}}^{+}|{\mathrm{CTA}}^{+}\right)\;=\mathrm{PPA}=n_{11}/n_{1}$
- $\omega_{10}\;=\mathrm{pr}\left(F1L\;{\mathrm{CDx}}^{+}\;\&\;{\mathrm{CTA}}^{‐}|{\mathrm{CTA}}^{‐}\right)=1‐\mathrm{NPA}=n_{01}/n_{0}$

The 95% CI was calculated using bootstrap simulation results (*n* = 5000).

#### 2.4.3 Sensitivity analysis

Imbalance in covariates (age, sex, baseline ECOG PS, race, smoking, baseline CNS metastases, histology) between F1L CDx‐evaluable and F1L CDx‐unevaluable groups was evaluated using non‐parametric Mann–Whitney (continuous variables) or Fisher–Freeman–Halton (Fisher’s exact) (categorical variables) tests. Association between covariates and F1L CDx result (detected vs. not detected) was evaluated via univariate logistic regression models, considering the above independent factors (excluding smoking) and clinical outcome.

To evaluate robustness of clinical efficacy estimates against missing F1L CDx results, unevaluable F1L CDx results were generated using multiple imputation with 50 imputed datasets, and PPA and NPA estimates were compared for three imputation models (Table S2). To assess sensitivity according to CTA type, PPA and NPA where then calculated and compared for the three CTA subgroups (Pharos, F1/F1Heme or other local tests; Table S3) using 50 imputed datasets and imputation model 3. Robustness of clinical endpoints was assessed using 50 imputed datasets and imputation model 1.

### 2.5 Resistance mutation analyses

Presence or absence of baseline primary resistance and post‐treatment acquired resistance mutations in driver proto‐oncogenes and the 10 most frequently altered genes was depicted graphically using oncoplots for each of the *NTRK*‐fp and *ROS1*‐fp populations, using matched pre‐treatment and PD plasma samples. Fisher’s exact test was performed for each gene to assess association between presence of a mutation and objective response (complete or partial response), with no multiplicity adjustments.

### 2.6 ctDNA fraction

To investigate whether ctDNA fraction influenced detection of fusions or secondary mutations in *NTRK1/3* or *ROS1,* ctDNA fraction was compared using a *t*‐test between samples in which a target aberration was detected and samples in which the corresponding aberration was not detected. Quantification of the ctDNA fraction was measured using two complementary methods: the proprietary tumour fraction estimator (TFE) and the maximum somatic allele frequency (MSAF) method [23]. The basis of TFE is a measure of tumour aneuploidy that incorporates observed deviations in coverage across the genome for a given sample. The calibration of calculated values for this metric is done to generate an estimate of the tumour fraction against a training set based on samples with well‐defined tumour fractions. MSAF is used when the TFE's ability to return an informative estimate is limited by lack of tumour aneuploidy. In the MSAF method, the allele fraction of all known somatic, likely somatic, and variant of unknown significance coding alterations by non‐PCR duplicate read pairs is used to estimate ctDNA fraction, with the exception of germline variants and variants in genes which are associated with clonal haematopoiesis.

## 3 Results

### 3.1 Plasma samples

**Fig. 1:**
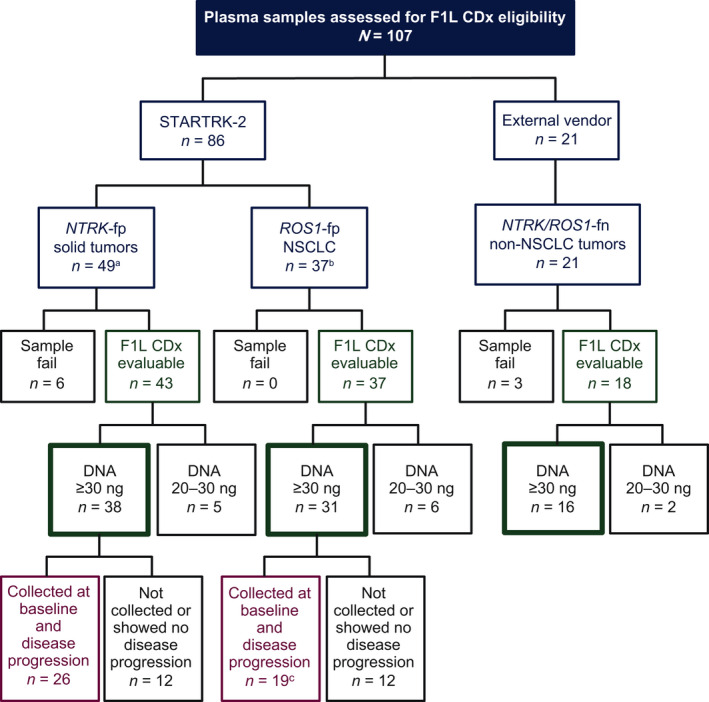
Flow chart of liquid biopsy samples used in the study. ^a^Five samples were unavailable for testing. ^b^16 samples were unavailable for testing. ^c^Two samples were excluded per FDA request.

Ninety‐eight pre‐treatment samples were evaluable by F1L CDx, of which 85 had DNA content ≥30 ng and were included in clinical bridging analyses (*NTRK*‐fp, *n* = 38; *ROS1*‐fp, *n* = 31; *NTRK/ROS1*‐fn, *n* = 16; Fig. 1). Forty‐five patients (*NTRK*‐fp, *n* = 26; *ROS1*‐fp, *n* = 19) had evaluable plasma samples (DNA content ≥30 ng) collected pre‐treatment and at/after, PD and were included in resistance mutation analyses (Fig. 1).

### 3.2 Clinical bridging analyses

#### 3.2.1 Concordance analysis

Concordance between the F1L CDx assay and CTAs for detection of *NTRK* and *ROS1* gene fusions is shown in Table S4. Agreement estimates between the F1L CDx assay and CTAs are summarized (Table 1). PPA between the F1L CDx assay and CTAs was 47.4% (95% CI: 31.0–64.2) for *NTRK* fusions and 64.5% (95% CI: 45.4–80.8) for *ROS1* fusions. PPV for the F1L CDx assay was 100% for *NTRK*‐fp and *ROS1*‐fp samples (Table 1). The assay detected *NTRK* and *ROS1* fusions regardless of whether the patients enrolled via local or central biomarker testing (Table S5), and across a variety of fusion partners (Table S6). There was a significant difference in sum of the longest diameters between tumours where *ROS1* was detected compared with those where no *ROS1* fusion was detected; this was not the case for *NTRK* fusions (Fig. S1).

**Table 1:** Performance of the F1L CDx assay for the detection of *NTRK* and *ROS1* gene fusions.

Parameter	*NTRK* gene fusion detection *N* = 85	*ROS1* gene fusion detection *N* = 85
PPA F1L CDx versus CTAs % (95% CI), *n*/*N*	47.4 (31.0–64.2); 18/38	64.5 (45.4–80.8); 20/31
NPA F1L CDx versus CTAs % (95% CI), *n*/*N*	100 (92.5–100.0); 47/47	100 (93.4–100); 54/54
PPV F1L CDx^a^ % (95% CI)	100 (82.4–100.0)	100 (83.9–100)
NPV F1L CDx^b^ % (95% CI)	99.8 (99.8–99.9)	99.6 (99.4–99.8)

^a^ The 95% CI of PPV was estimated by the Wilson score interval.

^b^ The 95% CI for NPV was estimated using the bootstrap method.

#### 3.2.2 Clinical efficacy analysis

Overall response rate (ORR) and DoR estimates according to CTA and F1L CDx results are presented for *NTRK*‐fp and *ROS1‐*fp cohorts (Table 2). Among patients with *NTRK*‐fp solid tumours, those classified as CTA^+^ F1L CDx^−^ or CTA^+^ F1L CDx unevaluable had lower ORR (ORR 55.0% and 43.8%, respectively) than those classified as CTA^+^ F1L CDx^+^ (ORR 72.2%). However, this was not statistically significant and was based on a small sample size therefore insufficient to conclude any differences between groups. As shown in Table 2 and Fig. S2, among patients with *NTRK*‐fp solid tumours and *ROS1‐*fp NSCLC, those classified as CTA^+^ F1L CDx^−^ or CTA^+^ F1L CDx unevaluable had longer median DoR (*NTRK‐*fp, 12.9 and 14.1 months, respectively; *ROS1*‐fp, 17.3 and 13.3 months, respectively) than those classified as CTA^+^ F1L CDx^+^ (*NTRK‐*fp, 9.2 months; *ROS1‐*fp, 5.6 months); however, this was only significant for patients with *ROS1‐*fp NSCLC (CTA^+^ F1L CDx^+^ vs. CTA^+^ F1L CDx^−^: *ROS1‐*fp, *P* = 0.009; *NTRK*‐fp, *P* = 0.434). F1L CDx clinical efficacy estimate, accounting for concordance between F1L CDx and CTA, gave an ORR of 72.2% (95% CI: 50.0–88.9) in the CTA^+^ F1L CDx^+^ *NTRK*‐fp solid tumour and *ROS1*‐fp NSCLC cohorts. As PPV was 100%, ORR for the overall F1L CDx^+^ population was equal to the ORR in the CTA^+^ F1L CDx^+^ cohort (72.2%).

**Table 2:** Clinical efficacy according to F1L CDx assay and CTA results. IQR, interquartile range.

	CTA^+^ ^a^	CTA^+^ F1L CDx evaluable				CTA^+^ F1L CDx unevaluable	*P*‐values^d^
		CTA^+^ F1L CDx^+^	CTA^+^ F1L CDx^−^	*P*‐values^c^	Total		
Patients with an *NTRK‐*fp solid tumour							
ORR (95% CI)	57.4 (43.2–70.8) *n* = 54	72.2 (46.5–90.3) *n* = 18	55.0 (31.5–76.9) *n* = 20	0.446	63.2 (47.3–76.6) *n* = 38	43.8 (19.8–70.1) *n* = 16	0.24
Median DoR, months (IQR)	10.4 (5.7–15.1) *n* = 54	9.2 (5.8–12.8) *n* = 18	12.9 (6.7–14.0) *n* = 20	0.434	9.3 (5.7–12.9) *n* = 38	14.1 (8.9–19.2) *n* = 16	0.40
Patients with *ROS1‐*fp NSCLC							
ORR (95% CI)	78.4 (64.8–88.7) *n* = 51^b^	72.2 (46.5–90.3) *n* = 18	72.7 (39.0–94.0) *n* = 11	1.00	72.4 (54.3–85.3) *n* = 29	86.4 (65.1–97.1) *n* = 22	0.31
Median DoR, months (IQR)	12.0 (5.6–17.2) *n* = 51	5.6 (3.5–11.4) *n* = 18	17.3 (13.9–18.8) *n* = 11	0.009	10.4 (3.7–17.2) *n* = 29	13.3 (8.5–16.4) *n* = 22)	0.31

^a^ ORR values in the CTA^+^ groups were derived in the ALKA‐372‐001/STARTRK‐1/STARTRK‐2 integrated analysis (May 2018 cut‐off) [17, 20].

^b^ Two *ROS1*‐fp patients were removed per FDA request.

^c^ *P*‐values derived with Wilcoxon rank‐sum test, for comparison between F1L CDx^+^ and F1L CDx^−^ groups.

^d^ *P*‐values derived from Fisher exact test for categorical factors between the F1L CDx evaluable set and the F1L CDx unevaluable set.

#### 3.2.3 Sensitivity analysis

For *NTRK*‐fp samples, covariates were balanced across evaluable (*n* = 38) and unevaluable (*n* = 16) F1L CDx groups (Table S7). For *ROS1*‐fp samples, covariate analysis indicated an unbalanced distribution in histology (*P* < 0.0001) and sex (*P* = 0.04) between evaluable (*n* = 29) and unevaluable (*n* = 22) F1L CDx groups (Table S8). F1L CDx outcome was significantly associated with histology (*P* = 0.03) using the *NTRK*‐fp evaluable set and with CNS lesions (*P* = 0.02) using the *ROS1*‐fp evaluable set (Table S9).

Estimations of PPA and NPA using imputation for F1L CDx unevaluable samples (*NTRK*‐fp 11/54; *ROS1*‐fp 22/51) were consistent across the three models, indicating robustness of the results (Table S10). PPA and NPA estimates according to CTA used were consistent across all three CTA subsets for *NTRK*‐fp samples. Variability was observed between Pharos and Others versus F1/F1 Haem for *ROS1*‐fp samples, but the latter population was too small for meaningful interpretation (Table S11). ORR estimations using imputation for *NTRK*‐fp (Table S12) and *ROS1*‐fp (Table S13) samples unevaluable by F1L CDx were similar to observed data.

### 3.3 Resistance mutation analyses

**Fig. 2:**
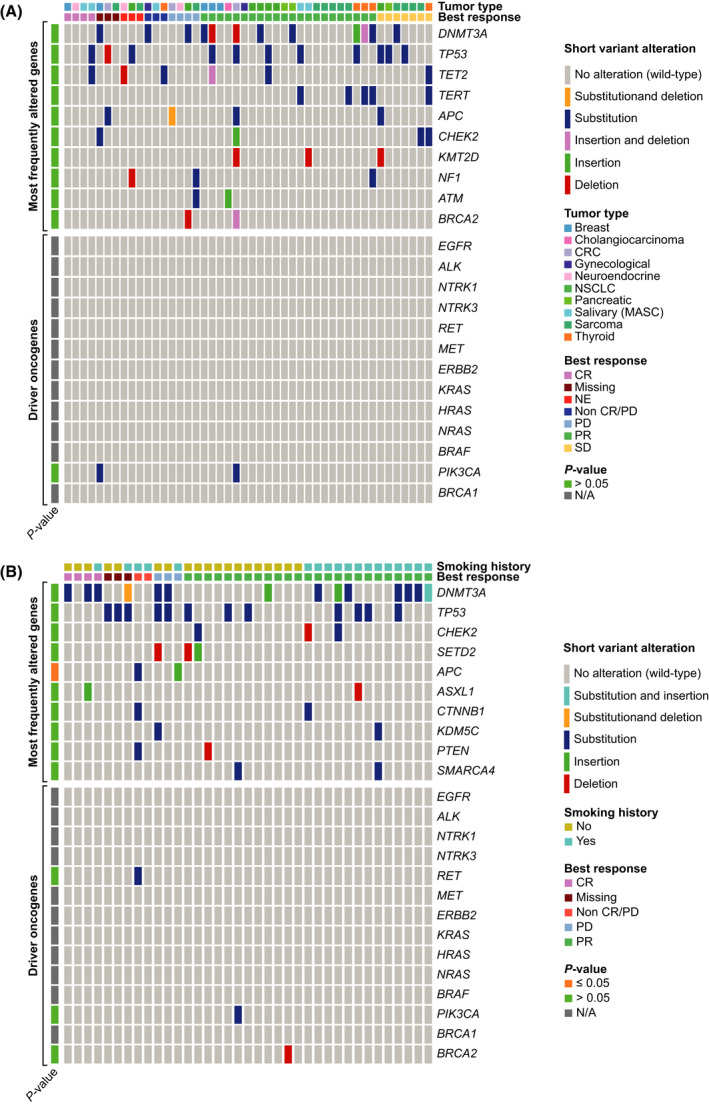
Primary resistance detected by F1L CDx, in patient ctDNA. Each column represents a single patient, with the genomic alterations indicated with non‐grey lines. Grey lines represent no alteration detected. Each sample was assayed a single time. Statistical analysis was carried out using Fisher’s exact test. (A) Patients with *NTRK*‐fp solid tumours; (B) Patients with *ROS1*‐fp NSCLC. CR, complete response; CRC, colorectal cancer; MASC, mammary analogue secretory carcinoma; N/A, not applicable; NE, non‐evaluable; PR, partial response; SD, stable disease.

Baseline co‐mutations present in *NTRK*‐fp (*n* = 46) and *ROS1*‐fp (*n* = 37) samples are shown in Fig. 2. In the *NTRK*‐fp cohort, the most frequently co‐mutated genes were *DNMT3A*, *TP53*, *TET2* and *TERT*, and no gene mutations were significantly associated with clinical response following entrectinib treatment. The most frequently co‐mutated genes in *ROS1*‐fp samples were *DNMT3A*, *TP53*, *CHEK2* and *SETD2*. For *ROS1*‐fp samples, a significant association was observed between presence of *APC* mutations and clinical non‐response following entrectinib treatment (*P* ≤ 0.05). Consistent with previous results [24, 25], neither of the *NTRK*‐fp or *ROS1*‐fp samples showed co‐mutation in other driver proto‐oncogenes (e.g. *EGFR*, *BRAF, KRAS*). We note that the detection of *DNMT3A*, *TET2* and *ASXL1* variants could also be explained by the common biological phenomenon of clonal haematopoiesis; however, as peripheral blood mononuclear cells were not collected and analysed as part of this study, we cannot definitely make this conclusion [26, 27].

**Fig. 3:**
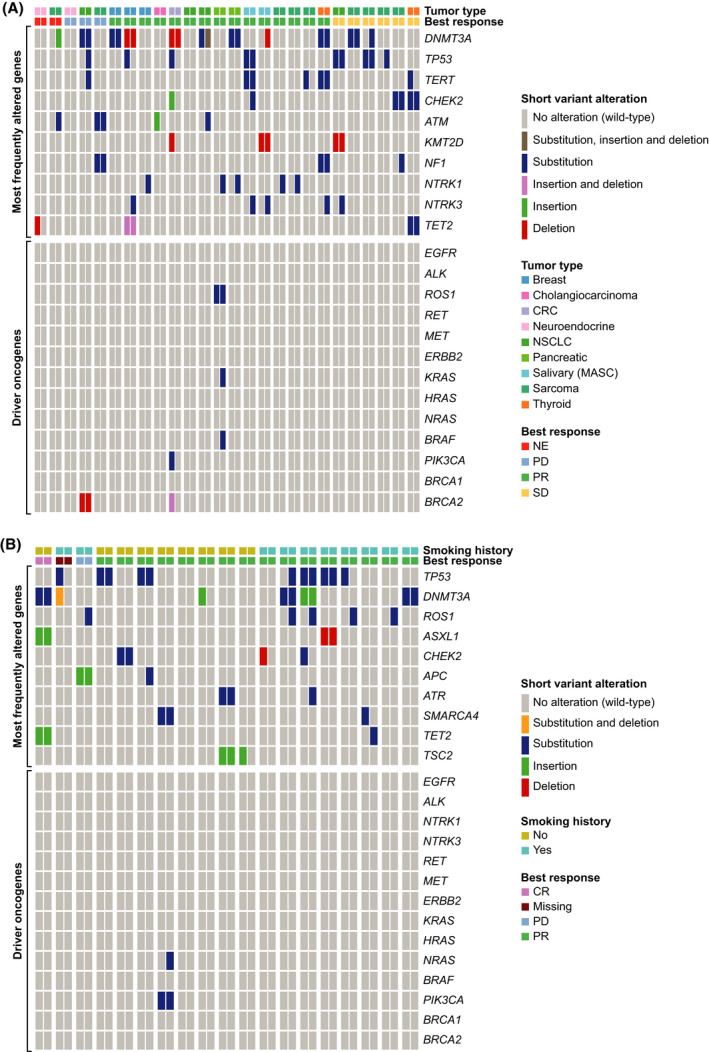
Acquired resistance mutations detected by F1L CDx, in patient ctDNA. Each paired column represents a single patient at two timepoints (pre‐treatment and end of study), with the genomic alterations indicated with a non‐grey line. Grey lines represent no alteration detected. Each sample was assayed a single time. Statistical analysis was carried out using Fisher’s exact test. (A) Patients with *NTRK*‐fp solid tumours; (B) Patients with *ROS1*‐fp NSCLC. CR, complete response; CRC, colorectal cancer; MASC, mammary analogue secretory carcinoma; NE, non‐evaluable; PR, partial response; SD, stable disease.

In the *NTRK*‐fp solid tumour cohort, acquired resistance mutations were observed across a range of tumour types (Fig. 3A). Ten patients (38%) had a detectable *NTRK* solvent front mutation at PD (*NTRK1^G595R^*, *n* = 5; *NTRK3^G623E/K/R^*, *n* = 5), which were not detected pre‐treatment. *BRAF^V600E^* and *KRAS^G12D^* mutations were detected in the PD sample from a patient with pancreatic cancer who had an initial partial response to entrectinib (DoR, 12.9 months). Notably, allele fractions of the *BRAF^V600E^* and *KRAS^G12D^* mutations were 0.51% and 5.15%, respectively, suggesting that these are independent resistance mechanisms arising from different metastatic lesions.

Fig. 3B presents acquired resistance mutations in pre‐treatment and post‐PD samples from the *ROS1*‐fp NSCLC cohort. Five patients (26%), four with tumours harbouring *CD74‐ROS1* fusions and one with a *SLC34A2‐ROS1* fusion‐positive tumour showed emergence of acquired *ROS1* resistance mutations (*ROS1^G2032R^*; *ROS1^F2004C/I^*) at PD, which were not present pre‐treatment.

An *NRAS^Q61K^* mutation was detected in the end of treatment sample from one patient with a partial response to entrectinib. Three patients with *NTRK*‐fp solid tumours and one with *ROS1*‐fp NSCLC had detectable mutations in *TP53* at/after PD that were not detected pre‐treatment.

### 3.4 ctDNA fraction

As detection of genomic alterations by ctDNA is highly dependent on the amount of tumour DNA present in blood, we calculated ctDNA fraction at baseline and end of study (Fig. S3). No significant association was observed between baseline *NTRK1/3* fusion detection or non‐detection and ctDNA fraction level (*P* = 0.152; Fig. S3A). Conversely, higher ctDNA fraction levels were associated with higher likelihood of *ROS1* fusion detection (*P* = 0.027; Fig. S3B). When comparing ctDNA fraction and detection levels of secondary *NTRK* or *ROS1* resistance mutations at end of study, no significant association was found between ctDNA fraction and detection of secondary point mutations in *NTRK1/3* (*P* = 0.479; Fig. S3C). However, higher ctDNA fraction was associated with higher likelihood of secondary *ROS1* fusion detection (*P* = 0.140; Fig. S3D).

## 4 Discussion

Availability of non‐invasive genomic profiling assays, such as F1L CDx, enables testing for clinically actionable oncogenic biomarkers. Repeated F1L CDx testing opens up the possibility of informed clinical decisions at all stages of a patient’s cancer management. Our analysis of pre‐treatment samples with F1L CDx yielded a 100% PPV, reflecting high confidence in detected *ROS1* and *NTRK* fusions. The clinical efficacy (ORR) estimate of 72.2% in CTA^+^ F1L CDx^+^ *NTRK*‐fp and *ROS1*‐fp cohorts trended to be higher than observed in the overall CTA^+^ *NTRK*‐fp cohort (57%) [20] and similar to the overall CTA^+^ *ROS1*‐fp cohort (77%) [17], supporting F1L CDx assay use in patients for whom tissue samples are unavailable or inadequate for NGS testing. Among patients with comparable NGS results, 38% with *NTRK*‐fp solid tumours and 26% with *ROS1*‐fp NSCLC acquired possible resistance mutations detected by F1L CDx with potential therapeutic implications, such as second‐generation tyrosine kinase inhibitor therapy.

Compared with predominantly tissue‐based CTAs (*n* = 83/85; 97.6%), the F1L CDx assay showed moderate PPAs of 47.4% and 64.5% for detection of *NTRK* and *ROS1* fusions, respectively, likely reflecting methodological differences (i.e. plasma vs. solid tissue and DNA‐ vs. RNA‐based CTAs, differences in diagnostic methodology between laboratories, tumour shedding variability among patients [28]). These values are consistent with plasma versus tissue PPA estimates for FDA‐approved plasma CDx assays, such as *Therascreen® PIK3CA RGQ PCR Kit* [54.6%; breast cancer (alpelisib)] and *cobas® EGFR Mutation Test v2* [≥58.7%; NSCLC (erlotinib, osimertinib, gefitinib)] [29, 30]. Additionally, given the aforementioned influence of differing methodology, our results are consistent with F1L CDx performance in detection of *PIK3CA* mutations in breast cancer (PPA, 71.7%) [31]. The high prevalence‐adjusted results are similar to those for F1L CDx detection of *ALK* rearrangements in NSCLC for alectinib treatment [12]. A 100.0% PPV for both *NTRK*‐fp and *ROS1*‐fp samples shows that, while F1L CDx may not detect all fusions present, positive results are very reliable and can rapidly inform clinical decisions. If fusions are not detected by F1L CDx because of patient variability in tumour type, stage, size, shedding, etc., further tissue‐based testing should be explored. The F1L CDx assay was able to detect gene fusions across a wide number of fusion partners, indicating no bias according to fusion partner.

A recent study also supports the detection of *NTRK* fusions across nine cancer types [32]. In this study, the authors report a PPV of 88% (CI 51–98%), which is similar to that observed here. However, direct comparison of these results should be taken with caution as the authors identified the *NTRK*‐fp samples using plasma and retrospectively tested for *NTRK* fusions with tissue. In addition, the NPV or ORR was not reported in the study.

High response rates were observed with entrectinib in patients classified as CTA^+^ F1L CDx^+^. This is consistent with a number of studies correlating liquid biopsy results and patient response to therapy [33, 34]. In the Phase III ALEX study of alectinib versus crizotinib in patients with advanced *ALK*‐fp NSCLC, although patients with high baseline cfDNA showed high response rates to targeted therapy, duration of response was much lower than observed for those with low baseline cfDNA [35]. This result was explained by baseline characteristics associated with higher tumour shedding (e.g. high tumour burden) and the authors concluded that baseline cfDNA acted as a prognostic factor for patient outcome. In our study, we observed a shorter median DoR in patients who were CTA^+^ F1L CDx^+^ versus CTA^+^ F1LCDx^−^, supporting that detection by F1L CDx could act as a prognostic factor for poorer patient outcome. However, this difference was only statistically significant in the *ROS1*‐fp cohort, likely reflecting the diverse cancer population of the *NTRK*‐fp cohort. CNS lesions are associated with more advanced disease [36] and, in our study, were also significantly associated with F1L CDx results in the *ROS1*‐fp cohort. Although the small sample size makes it difficult to draw definitive conclusions, these findings support the hypothesis that the likelihood of detecting gene fusions by F1L CDx may be higher in samples from patients with higher tumour burden and tumour shedding [37]. Similarly, within the *ROS1*‐fp cohort, ctDNA fraction, which may be a surrogate for tumour size and/or shedding, was higher in patients with a detected baseline fusion.

**Fig. 4:**
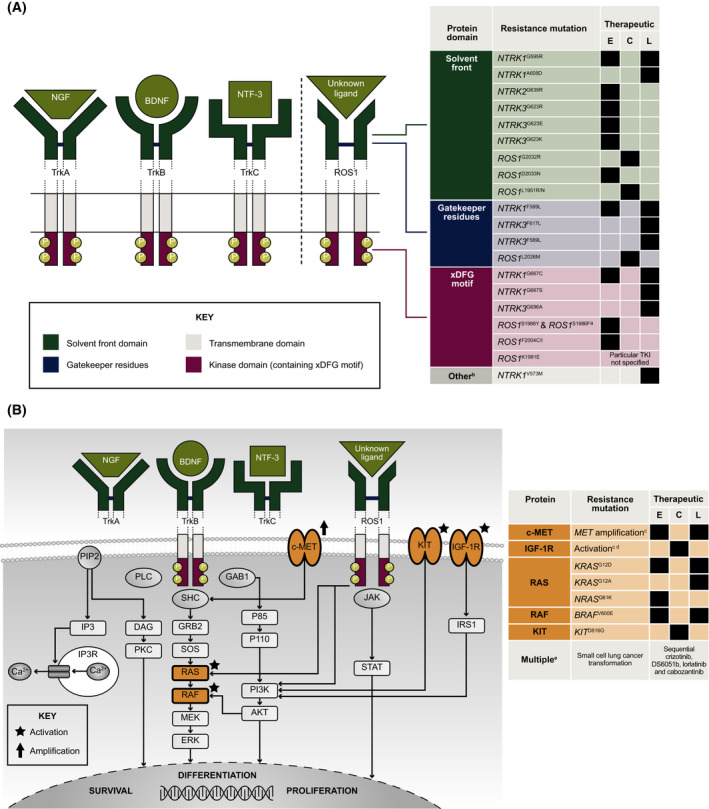
Mechanisms of acquired resistance to TKIs in *NTRK*‐fp solid tumours and *ROS1‐*fp NSCLC: (A) On‐target resistance; (B) Off‐target resistance. Black square indicates clinical resistance observed following treatment with therapeutic. ^a^Dual mutation. ^b^Functional domain/structure not reported/unknown. ^c^Specific mutation unknown/not reported. ^d^Reported in patients with *ALK* fusion‐positive lung cancer only. ^e^Complete genomic profile of transformed SCLC not fully elucidated. ALK, anaplastic lymphoma kinase; BDNF, brain‐derived neurotrophic factor; C, crizotinib; DFG, Asp‐Phe‐Gly motif; E, entrectinib; KIT, KIT proto‐oncogene, receptor tyrosine kinase; KRAS, Kirsten rat sarcoma virus; L, larotrectinib; MET, mesenchymal epithelial transition; NGF, nerve growth factor beta; NRAS, neuroblastoma RAS viral (v‐ras) oncogene homolog; NTF‐3, Neurotrophin‐3; TKI, tyrosine kinase inhibitor; Trk, tropomyosin receptor kinase.

As is common in targeted therapy, emergence of mutations associated with re‐activation of the target gene [e.g. *ROS1* or *NTRK* (Fig. 4)] or the downstream mitogen‐activated protein kinase (MAPK) pathway have been described at progression, including entrectinib and larotrectinib [38, 39]. *In* *vitro* resistance studies have also predicted *KRAS/NRAS* mutations to drive resistance to *ROS1* inhibitors [40]. In the current study, acquired *MAPK* mutations were observed in one patient in each of the *NTRK*‐fp and *ROS1*‐fp cohorts, providing further evidence to support MAPK reactivation as a key mechanism of entrectinib resistance. One patient with pancreatic cancer showed mutations in both the *KRAS* and *BRAF* genes, despite previous evidence that these mutations are mutually exclusive due to overlapping functionality [41]. We hypothesize that *KRAS* and *BRAF* mutations are unlikely to develop within the same lesion given differences in allele frequencies and may reflect tumour heterogeneity detected by the F1L CDx assay. A relatively high frequency of *TP53* mutations was observed in *NTRK*‐fp and *ROS1*‐fp samples, which are a hallmark of a hypermutation phenotype [42, 43]. Their presence may therefore suggest a hypermutation phenotype rather than site‐specific mutation in these fusion‐positive tumours.

Within the *NTRK*‐fp cohort, we did not find co‐mutations present at baseline that were significantly associated with clinical benefit following entrectinib treatment. We hypothesize that this may be partly due to the heterogeneity in tumour type in our sample for this cohort, as co‐mutations may vary between tumour types and may therefore have confounded this result. In the *ROS1* cohort, the *APC* gene was associated with poor clinical response to entrectinib; however, this was based on a small number of cases and requires further study in a larger population.

This study was retrospective and has a relatively small sample size, reflecting the rarity of *NTRK* and *ROS1* fusions. However, samples and comprehensive clinical data were collected as part of a prospective, global clinical trial leading to the approval of entrectinib. The availability of matched samples before treatment initiation and following progression also facilitated detailed examination of resistance mutations arising following entrectinib treatment. The current analyses thus comprise a unique dataset, due to the rarity of the tumour types studied and the scarcity of prospective data on resistance mechanisms.

## 5 Conclusion

Patients with *NTRK*‐fp solid tumours or *ROS1*‐fp NSCLC detected by CTAs and F1L CDx assay demonstrate high response rates to entrectinib, which can complement tissue‐based testing for the identification of patients who may benefit from entrectinib treatment. Additionally, the F1L CDx assay can identify acquired resistance mechanisms in patients who experience progression following entrectinib. The F1L CDx assay can therefore be used to inform clinical decisions at all stages of the patient journey from diagnosis to disease progression, to ensure rapid access to targeted treatment in fusion‐positive patients, including patients for whom tissue biopsies are contraindicated or where tumour samples are limited. Assessment at progression will support initiation of second‐generation TRK or ROS1 inhibitors, or potential combination regimens, if resistance mutations are detected.

## Conflict of interest

The authors declare the following competing financial interests: RD has received consultancy/advisory fees from F. Hoffmann‐La Roche Ltd, Foundation Medicine Inc., Pfizer, AstraZeneca, Celon Pharma, Bristol‐Myers Squibb, Merck, MSD, Regeneron, Takeda, Seattle Genetics, Novartis; and has received travel or accommodation expenses from F. Hoffmann‐La Roche Ltd and AstraZeneca. TH, VC, CW, CWC, BS, and TR are employees of Genentech, Inc. AD reports honoraria/advisory fees from Ignyta/Genentech, Inc./F. Hoffmann‐La Roche Ltd, Loxo/Bayer/Lilly, Takeda/Ariad/Millenium, TP Therapeutics, AstraZeneca, Pfizer, Blueprint Medicines, Helsinn, Beigene, BergenBio, Hengrui Therapeutics, Exelixis, Tyra Biosciences, Verastem, MORE Health, Abbvie, 14ner/Elevation Oncology, Remedica Ltd, ArcherDX, Monopteros, Novartis, EMD Serono, Melendi, Liberum, Repare RX, Nuvalent, Merus; research grant/funding (institution) from Pfizer, Exelixis, GlaxoSmithKlein, Teva, Taiho, PharmaMar; research grant (self) from Foundation Medicine; royalties from Wolters Kluwer; expenses from Merck, Puma, Merus, Boehringer Ingelheim; CME honoraria from Medscape, OncLive, PeerVoice, Physicians Education Resources, Targeted Oncology, Research to Practice, Axis, Peerview Institute, Paradigm Medical Communications, WebMD, MJH Life Sciences, Med Learning, Imedex, Answers in CME, Clinical Care Options. RCD declares consulting fees from Ignyta, Genentech, Inc./F. Hoffmann‐La Roche Ltd, AstraZeneca, Anchiano, and Rain Therapeutics; royalties or licensing fees for intellectual property from Ignyta, Abbott Molecular, Genentech, Inc./F. Hoffmann‐La Roche Ltd, Foundation Medicine, Black Diamond, Pearl River, Voronoi, Takeda, Scorpion, and Rain Therapeutics; stock ownership in Rain Therapeutics; is an employee of Rain Therapeutics. FB reports consulting/advisory role for F. Hoffmann‐La Roche Ltd/Genentech, Inc., Pfizer, Novartis, Pierre Fabre, Bristol‐Myers Squibb, AstraZeneca/MedImmune, Boehringer Ingelheim, Lilly, Merck Serono, MSD Oncology, Takeda, Bayer; travel/accommodation/expenses from F. Hoffmann‐La Roche Ltd/Genentech, Inc., Bristol‐Myers Squibb, AstraZeneca/MedImmune, MSD Oncology; honoraria from F. Hoffmann‐La Roche Ltd/Genentech, Inc., Pfizer, Pierre Fabre, AstraZeneca, Bristol‐Myers Squibb, Boehringer Ingelheim, Lilly, Novartis, Pierre Fabre, Merck Serono, MSD Oncology, Takeda, Bayer; research funding (institution) from F. Hoffmann‐La Roche Ltd/Genentech, Inc., AstraZeneca/MedImmune, Bristol‐Myers Squibb, Pierre Fabre, Abbvie, Amgen, Bayer, Boehringer Ingelheim, Eisai, Lilly, Ipsen, Innate Pharma, Novartis, Merck Serono, MSD Oncology, Pfizer, Sanofi/Aventis, Takeda. TRW is an employee of Genentech, Inc., and has stock and ownership interest in F. Hoffmann‐La Roche Ltd. KW was an employee of Foundation Medicine, Inc. at the time of the study but is now an employee of the US Food and Drug Administration. JS, LD, RW, and ML are employees of Foundation Medicine, Inc.

## Author contributions

TH, RW, CW and TRW were involved in conception and design of the analysis. TH, CW and TRW were involved in conduct of the analysis. All authors were involved in data interpretation/analysis. All authors were involved in manuscript drafting, writing and editing. All authors approved the manuscript for submission.

## Supporting information


**Fig. S1**. Differences in sum of the longest diameters between tumours where *ROS1* or *NTRK* fusions were detected by F1L CDx versus those with no detected fusion.
**Fig. S2**. Duration of response in patients with *NTRK* fusion‐positive solid tumours and *ROS1* fusion‐positive NSCLC by ctDNA status.
**Fig. S3**. F1L CDx detection of primary and secondary mutations in *NTRK1/2/3* and *ROS1* fusion‐positive samples. (A) Primary *NTRK1/2/3* fusion. (B) Primary *ROS1* fusion. (C) Secondary *NTRK1/2/3* mutation. (D) Secondary *ROS1* mutation.
**Table S1**. Number of patients by *NTRK*‐fp solid tumour type within each CTA^+^ subgroup.
**Table S2**. Imputation models used in sensitivity analyses.
**Table S3**. CTAs used to confirm the presence of *NTRK* or *ROS1* gene fusions in tumour samples from patients enrolled in STARTRK‐2 with valid F1L CDx results.
**Table S4**. Concordance between the F1L CDx assay and CTAs for the detection of *NTRK* and *ROS1* gene fusions.
**Table S5**. F1L CDx *NTRK* and *ROS1* fusion detection and method of enrolment (central vs local).
**Table S6**. List and prevalence of *NTRK* and *ROS1* fusion partners detected by the F1L CDx assay.
**Table S7**. Demographic and clinical characteristics for patients with *NTRK*‐fp solid tumours.
**Table S8**. Demographic and clinical characteristics for patients with *ROS1*‐fp NSCLC.
**Table S9**. Univariate logistic regression model evaluating relationships between covariates and F1L CDx test results (Positive vs Negative).
**Table S10**. Sensitivity of PPA and NPA according to baseline characteristics, Pharos result and response.
**Table S11**. Sensitivity of PPA and NPA according to CTA used.
**Table S12**. Robustness of ORR using imputation Model 1 for *NTRK*‐fp samples.
**Table S13**. Robustness of ORR using imputation Model 1 for *ROS1‐*fp samples.

## Data Availability

For eligible studies, qualified researchers may request access to individual patient level clinical data through a data request platform. At the time of writing, this request platform is Vivli: https://vivli.org/ourmember/roche/. For up‐to‐date details on Roche's Global Policy on the Sharing of Clinical Information and how to request access to related clinical study documents, see here: https://go.roche.com/data_sharing. Anonymized records for individual patients across more than one data source external to Roche cannot, and should not, be linked due to a potential increase in risk of patient re‐identification.
