# Supplementary material for: Pre‐ and post‐treatment blood‐based genomic landscape of patients with ROS1 or NTRK fusion‐positive solid tumours treated with entrectinib
Source: Mol Oncol. 2022 Apr 22;16(10):2000–14. doi: 10.1002/1878-0261.13214 (PMC9120896; doi:10.1002/1878-0261.13214)
Supplement: Supplementary file 1 — Fig. S1. Differences in sum of the longest diameters between tumours where ROS1 or NTRK fusions were detected by F1L CDx versus those with no detected fusion. Fig. S2. Duration of response in patients with NTRK fusion‐positive solid tumours and ROS1 fusion‐positive NSCLC by ctDNA status. Fig. S3. F1L CDx detection of primary and secondary mutations in NTRK1/2/3 and ROS1 fusion‐positive samples. (A) Primary NTRK1/2/3 fusion. (B) Primary ROS1 fusion. (C) Secondary NTRK1/2/3 mutation. (D) Secondary ROS1 mutation. Table S1. Number of patients by NTRK‐fp solid tumour type within each CTA+ subgroup. Table S2. Imputation models used in sensitivity analyses. Table S3. CTAs used to confirm the presence of NTRK or ROS1 gene fusions in tumour samples from patients enrolled in STARTRK‐2 with valid F1L CDx results. Table S4. Concordance between the F1L CDx assay and CTAs for the detection of NTRK and ROS1 gene fusions. Table S5. F1L CDx NTRK and ROS1 fusion detection and method of enrolment (central vs local). Table S6. List and prevalence of NTRK and ROS1 fusion partners detected by the F1L CDx assay. Table S7. Demographic and clinical characteristics for patients with NTRK‐fp solid tumours. Table S8. Demographic and clinical characteristics for patients with ROS1‐fp NSCLC. Table S9. Univariate logistic regression model evaluating relationships between covariates and F1L CDx test results (Positive vs Negative). Table S10. Sensitivity of PPA and NPA according to baseline characteristics, Pharos result and response. Table S11. Sensitivity of PPA and NPA according to CTA used. Table S12. Robustness of ORR using imputation Model 1 for NTRK‐fp samples. Table S13. Robustness of ORR using imputation Model 1 for ROS1‐fp samples. [file MOL2-16-2000-s001.docx]

**Supplementary materials**

**Table S1** Number of patients by *NTRK*-fp solid tumor type within each CTA+ subgroup

|  |  | CTA+ F1L CDx evaluable (*n* = 38) | | |  |
| --- | --- | --- | --- | --- | --- |
| Tumor type, *n* | Total CTA+  (*n* = 54) | CTA+ F1L CDx+  (*n* = 18) | CTA+ F1L CDx-  (*n* = 20) | Total  (*n* = 38) | CTA+ F1L CDx unevaluable  (*n* = 16) |
| Breast | 6 | 2 | 2 | 4 | 2 |
| GI – CRC | 4 | 2 | 0 | 2 | 2 |
| GI – non-CRC | 4 | 3 | 0 | 3 | 1 |
| Gynecological | 2 | 0 | 1 | 1 | 1 |
| Head and Neck | 12 | 4 | 3 | 7 | 5 |
| Neuroendocrine | 3 | 1 | 2 | 3 | 0 |
| Sarcoma | 13 | 2 | 9 | 11 | 2 |
| Thoracic | 10 | 4 | 3 | 7 | 3 |

CRC, colorectal cancer; CTA, clinical trial assays; F1L CDx, FoundationOne Liquid CDx; fp, fusion-positive; GI, gastrointestinal; NTRK, neurotrophic tyrosine receptor kinase.

**Table S2** Imputation models used in sensitivity analyses

| **Model** | **Independent factors specification** | |
| --- | --- | --- |
|  | ***NTRK* fusion sub-population** | ***ROS1* fusion sub-population** |
| Model 1 | logit(p)^a^ ~ age, sex, race, ECOG PS, CNS metastases at baseline, histology^b^, Pharos result | logit(p)^a^ ~ age, sex, race, ECOG PS, CNS metastases at baseline, Pharos result |
| Model 2 | logit(p)^a^ ~ Treatment response, Pharos result | logit(p)^a^ ~ Treatment response, Pharos result, CNS metastases at baseline |
| Model 3 | logit(p)^a^ ~ Treatment response | logit(p)^a^ ~ Pharos result |

^a^p is the probability of a positive F1L CDx outcome.
^b^Histology was modelled as a binary variable with adenocarcinoma and non-adenocarcinoma categories.
CNS, central nervous system; ECOG, Eastern Cooperative Oncology Group performance score; F1L CDx, FoundationOne Liquid CDx; NTRK, neurotrophic tyrosine receptor kinase; ROS1, ROS proto-oncogene 1.

**Table S3** CTAs used to confirm the presence of *NTRK* or *ROS1* gene fusions in tumor samples from patients enrolled in STARTRK-2 with valid F1L CDx results

| **Central / local testing** | **Sample type** | **Samples identified as *NTRK*-fp, *n*/*N*** | **Samples identified as *ROS1*-fp, *n*/*N*** |
| --- | --- | --- | --- |
| Local | DNA | 15/43 | 7/35 |
| Local (plasma) | DNA | 2/43 |  |
| Local | DNA & RNA | 3/43 | 2/35 |
| Central (Trailblaze Pharos™) | RNA | 18/43 | 23/35 |
| Local | RNA | 5/43 | 3/35 |

CTA, clinical trial assay; F1L CDx, FoundationOne Liquid CDx; fp, fusion-positive; NTRK, neurotrophic tyrosine receptor kinase; ROS1, ROS proto-oncogene 1.

**Table S4** Concordance between the F1L CDx assay and CTAs for the detection of *NTRK* and *ROS1* gene fusions

| ***NTRK* gene fusion** | **CTAs** | | | |
| --- | --- | --- | --- | --- |
| **F1L CDx** |  | **Detected** | **Not detected** | **Total** |
|  | **Detected** | 18 | 0 | 18 |
|  | **Not detected** | 20 | 47 | 67 |
|  | **Total** | 38 | 47 | 85 |
| ***ROS1* gene fusion** | **CTAs** | | | |
| **F1L CDx** |  | **Detected** | **Not detected** | **Total** |
|  | **Detected** | 20 | 0 | 20 |
|  | **Not detected** | 11 | 54 | 65 |
|  | **Total** | 31 | 54 | 85 |

CTA, clinical trial assays; F1L CDx, FoundationOne Liquid CDx; NTRK, neurotrophic tyrosine receptor kinase; ROS1, ROS proto-oncogene 1.

**Table S5** F1L CDx *NTRK* and *ROS1* fusion detection and method of enrollment (central vs local)

| *NTRK* gene fusion | | | |
| --- | --- | --- | --- |
| CTA tissue enrollment | F1L unevaluable  (*n* = 16) | F1L fusion detected  (*n* = 18) | F1L fusion not detected  (*n* = 20) |
| Central | 5 | 8 | 8 |
| Local | 11 | 10 | 12 |
| *ROS1* gene fusion | | | |
| CTA tissue enrollment | F1L unevaluable  (*n* = 22) | F1L fusion detected  (*n* = 18) | F1L fusion not detected  (*n* = 11) |
| Central | 3 | 12 | 8 |
| Local | 19 | 6 | 3 |

CTA, clinical trial assays; F1L CDx, FoundationOne Liquid CDx; NTRK, neurotrophic tyrosine receptor kinase; ROS1, ROS proto-oncogene 1.

**Table S6** List and prevalence of *NTRK* and *ROS1* fusion partners detected by the F1L CDx assay

| **Status per F1L CDx assessment** | **Fusion partner per CTA** | | | | | | | | | | | |
| --- | --- | --- | --- | --- | --- | --- | --- | --- | --- | --- | --- | --- |
|  | ***NTRK*-fp samples** | | | | | | ***ROS1*-fp samples** | | | | | |
|  | *ETV6-NTRK3* | *TPM3-NTRK1* | *TPR-NTRK1* | *LMNA-NTRK1* | All other fusions^a^ | **Total** | *CD74-ROS1* | *EZR-ROS1* | *SLC34A2-ROS1* | *SDC4-ROS1* | *TPM3-ROS1* | **Total** |
| Detected, *n* (%) | 7 (36.8) | 1  (5.3) | 3 (15.8) | 1  (5.3) | 7  (36.8) | **19** | 11 (57.9) | 3 (15.8) | 2  (10.5) | 3 (15.8) | 0 | **19** |
| Not detected, *n* (%) | 12 (50.0) | 2  (8.3) | 1  (4.2) | 0 | 9  (37.5) | **24** | 7 (43.8) | 0 | 5  (31.3) | 2 (12.5) | 2 (12.5) | **16** |

^a^Detected: *CD74-NTRK1, CDC42BPA-NTRK1, CGN-NTRK1, EPS15L1-NTRK1, PEAR1-NTRK1, PLEKHA6-NTRK1, TRIM33-NTRK1*;
Not-detected: *AKAP13-NTRK3 EML4-NTRK3, FAM19A2-NTRK3, KIF7-NTRK3, PDIA3-NTRK1, PEAR1-NTRK1, RBPMS-NTRK3, SQSTM1-NTRK1, SQSTM1-NTRK2*.
CD74, Cluster of Differentiation 74; CTA, clinical trial assays; ETV6, transcription factor ETV6; EZR, ezrin; F1L CDx, FoundationOne Liquid CDx; fp, fusion-positive; LMNA, lamin A/C; NTRK, neurotrophic tyrosine receptor kinase; ROS1, ROS proto-oncogene 1; SDC4, syndecan 4; SLC34A2, sodium-dependent phosphate transport protein 2B; TPM3, tropomyosin 3; TPR, translocated promoter region.

**Table S7** Demographic and clinical characteristics for patients with *NTRK*-fp solid tumors

| **Covariates, *n* (%)** | **Total CTA+ (*N* = 54)** | **CTA+ F1L CDx evaluable (*n* = 38)** | | | **CTA+ F1L CDx unevaluable (*n* = 16)** | ***P*-values (CTA+ F1L CDx evaluable versus CTA+ F1L CDx unevaluable)** |
| --- | --- | --- | --- | --- | --- | --- |
|  |  | **CTA+ F1L CDx+ (*n* = 18)** | **CTA+ F1L CDx- (*n* = 20)** | **Total (*n* = 38)** |  |  |
| ORR, % (95% CI) | 57.4 (43.2–70.8) | 72.2  (46.5–90.3) | 55.0  (31.5–76.9) | 63.2  (47.3–76.6) | 43.8  (19.8–70.1) | 0.24 |
| Age, years |  |  |  |  |  | 0.79^a^ |
| Mean (SD) | 57.0 (14.4) | 57.2 (12.6) | 55.6 (17.1) | 56.3 (15.0) | 55.8 (13.5) |  |
| Sex, *n* (%) |  |  |  |  |  | 1.00 |
| Male | 22 (40.7) | 8 (44.4) | 8 (40.0) | 16 (42.1) | 6 (37.5) |  |
| Female | 32 (59.3) | 10 (55.6) | 12 (60.0) | 22 (57.9) | 10 (62.5) |  |
| ECOG PS, *n* (%) |  |  |  |  |  | 0.76 |
| 0 | 23 (42.6) | 5 (27.8) | 10 (50.0) | 15 (39.5) | 8 (50.0) |  |
| 1 | 25 (46.3) | 11 (61.1) | 7 (35.0) | 18 (47.4) | 7 (43.7) |  |
| 2 | 6 (11.1) | 2 (11.1) | 3 (15.0) | 5 (13.2) | 1 (6.3) |  |
| Race, *n* (%) |  |  |  |  |  | 1.00 |
| Asian | 7 (13.0) | 4 (22.2) | 1 (5.0) | 5 (13.2) | 2 (12.5) |  |
| White | 43 (79.6) | 13 (72.2) | 19 (95.0) | 32 (84.2) | 11 (68.8) |  |
| NR | 4 (7.4) | 1 (5.6) | 0 | 1 (2.6) | 3 (18.8) |  |
| Smoking history, *n* (%) |  |  |  |  |  | 1.00 |
| Current | 6 (11.1) | 3 (16.7) | 2 (10.0) | 5 (13.2) | 1 (6.3) |  |
| Former | 17 (31.4) | 7 (38.9) | 5 (25.0) | 12 (31.6) | 5 (31.3) |  |
| NR | 31 (57.4) | 8 (44.4) | 13 (65.0) | 21 (55.3) | 10 (62.5%) |  |
| CNS metastases at baseline, *n* (%) |  |  |  |  |  | 1.00 |
| Yes | 12 (22.2) | 6 (33.3) | 3 (15.0) | 9 (23.7) | 3 (18.8) |  |
| No | 42 (77.8) | 12 (66.7) | 17 (85.0) | 29 (76.3) | 13 (81.3) |  |
| Histology, *n* (%) |  |  |  |  |  | 0.23^b^ |
| Adenocarcinoma | 18 (33.3) | 9 (50.0) | 3 (15.0) | 12 (31.6) | 6 (37.5) |  |
| Carcinomas with pleomorphic, sarcomatoid | 2 (3.7) | 0 | 1 (5.0) | 1 (2.6) | 1 (6.3) |  |
| Neuroendocrine | 3 (5.6) | 1 (5.6) | 2 (10.0) | 3 (7.9) | 0 (0.0) |  |
| Spindle cell | 6 (11.1) | 1 (5.6) | 4 (20.0) | 5 (13.2) | 1 (6.3) |  |
| Squamous-cell carcinoma | 2 (3.7) | 1 (5.6) | 0 | 1 (2.6) | 1 (6.3) |  |
| Other | 18 (33.3) | 6 (33.3) | 9 (45.0) | 15 (39.5) | 3 (18.8) |  |
| Unclassified/undifferentiated carcinoma | 3 (5.6) | 0 | 1 (5.0) | 1 (2.6) | 2 (12.5) |  |
| NR | 2 (3.7) | 0 | 0 | 0 (0.0) | 2 (12.5) |  |
| Numbers of prior lines of systemic therapies, *n* (%) |  |  |  |  |  | 1.00 |
| < 2 | 30 (55.6) | 9 (50.0) | 12 (60.0) | 21 (55.3) | 9 (56.3) |  |
| ≥ 2 | 23 (42.6) | 9 (50.0) | 8 (40.0) | 17 (44.7) | 6 (37.5) |  |
| Missing | 1 (1.9) | 0 (0) | 0 (0) | 0 (0) | 1 (6.3) |  |

^a^*P*-value derived with Welch two sample t test (normality evaluated using Shapiro-Wilk test)
^b^*P*-value derived by comparing Adenocarcinoma, Others and combined remaining categories
CI, confidence interval; CNS, central nervous system; CTA, clinical trial assay; ECOG PS, Eastern Cooperative Oncology Group performance status; F1L CDx, FoundationOne Liquid CDx: fp, fusion-positive; NR, not reported; NTRK, neurotrophic tyrosine receptor kinase; ORR, objective response rate; SD, standard deviation.

**Table S8** Demographic and clinical characteristics for patients with *ROS1*-fp NSCLC

| **Covariates, *n* (%)** | **CTA+ (*N* = 51)** | **CTA+ F1L CDx evaluable (*n* = 29)** | | | **CTA+ F1L CDx unevaluable (*n* = 22)** | ***P*-values (CTA+ F1LCDx evaluable versus CTA+ F1L CDx unevaluable)** |
| --- | --- | --- | --- | --- | --- | --- |
|  |  | **CTA+ F1L CDx+ (*n* = 18)** | **CTA+ F1L CDx- (*n* = 11)** | **Total  (*n* = 29)** |  |  |
| ORR, % (95% CI) | 78.4 (64.8–88.7) | 72.2  (49.1–87.5) | 72.7  (39.0–94.0) | 72.4  (54.3–85.3) | 86.4 (65.1–97.1) | 0.31 |
| Age, years |  |  |  |  |  | 0.85^a^ |
| Mean (SD) | 53.2 (11.2) | 51.8 (12.9) | 51.9 (10.0) | 51.8 (11.7) | 55.0 (10.5) |  |
| Sex, *n* (%) |  |  |  |  |  | 0.04 |
| Male | 17 (33.3) | 4 (22.2) | 2 (18.2) | 6 (20.7) | 11 (50.0) |  |
| Female | 34 (66.6) | 14 (77.8) | 9 (81.8) | 23 (79.3) | 11 (50.0) |  |
| ECOG PS, *n* (%) |  |  |  |  |  | 0.32 |
| 0 | 19 (37.3) | 4 (22.2) | 5 (45.5) | 9 (31.0) | 10 (45.5) |  |
| 1 | 26 (51.0) | 9 (50.0) | 6 (54.5) | 15 (51.7) | 11 (50.0) |  |
| 2 | 6 (11.7) | 5 (27.8) | 0 | 5 (17.2) | 1 (4.5) |  |
| Race, n (%) |  |  |  |  |  | 0.35 |
| Asian | 19 (37.3) | 8 (44.4) | 3 (27.3) | 11 (37.9) | 8 (36.4) |  |
| Black/African American | 3 (5.9) | 2 (11.1) | 1 (9.1) | 3 (10.3) | 0 (0.0) |  |
| White | 29 (56.9) | 8 (44.4) | 7 (63.6) | 15 (51.7) | 14 (63.6) |  |
| Smoking history,  *n* (%) |  |  |  |  |  | 1.00 |
| Current | 2 (3.9) | 1 (5.6) | 0 | 1 (3.4) | 1 (4.6) |  |
| Former | 20 (39.2) | 8 (44.4) | 5 (45.5) | 13 (44.8) | 7 (31.8) |  |
| NR | 29 (56.9) | 9 (50.0) | 5 (54.5) | 15 (51.7) | 14 (63.6) |  |
| CNS metastases at baseline, *n* (%) |  |  |  |  |  | 0.56 |
| Yes | 22 (43.1) | 12 (66.7) | 2 (18.2) | 14 (48.3) | 8 (36.4) |  |
| No | 29 (56.9) | 6 (33.3) | 9 (81.8) | 15 (51.7) | 14 (63.6) |  |
| Histology, *n* (%) |  |  |  |  |  | < 0.0001 |
| Adenocarcinoma | 34 (66.7) | 17 (94.4) | 11 (100.0) | 28 (96.6) | 6 (27.3) |  |
| Carcinomas with pleomorphic, sarcomatoid or sarcomatous elements | 1 (2.0) | 1 (5.6) | 0 | 1 (3.4) | 0 |  |
| Other | 9 (17.6) | 0 | 0 | 0 | 9 (40.9) |  |
| NR | 7 (13.7) | 0 | 0 | 0 | 7 (31.8) |  |
| Numbers of prior lines of systemic therapies, *n* (%) |  |  |  |  |  | 0.40 |
| < 2 | 34 (66.7) | 13 (72.2) | 9 (81.8) | 22 (75.9) | 12 (54.6) |  |
| ≥ 2 | 8 (15.7) | 5 (27.8) | 2 (18.2) | 7 (24.1) | 1 (4.6) |  |
| Missing | 9 (17.7) | 0 (0) | 0 (0) | 0 (0) | 9 (40.9) |  |

^a^*P*-value derived with Welch two sample t test (normality evaluated using Shapiro-Wilk test)
CI, confidence interval; CNS, central nervous system; CTA, clinical trial assay; ECOG PS, Eastern Cooperative Oncology Group performance status; F1L CDx, FoundationOne Liquid CDx; fp, fusion-positive; NR, not reported; NSCLC, non-small cell lung cancer; ORR, objective response rate; ROS1, ROS proto-oncogene 1; SD, standard deviation.

**Table S9** Univariate logistic regression model evaluating relationships between covariates and F1L CDx test results (Positive vs Negative)^a^

| **Covariates** | **Estimate of odds ratio** | ***P*-value** |
| --- | --- | --- |
| **Patients with an *NTRK-*fp solid tumor** | | |
| Age | 1.01 | 0.74 |
| Sex-Male | 1.20 | 0.78 |
| Race-White | 0.17 | 0.13 |
| ECOG PS |  |  |
| 1 | 3.14 | 0.12 |
| 2 | 1.33 | 0.79 |
| CNS metastases at baseline | 2.83 | 0.19 |
| Histology-Adenocarcinoma to Others^b^ | 5.68 | 0.03 |
| Individual response | 2.13 | 0.28 |
| **Patients with *ROS1-*fp NSCLC** | | |
| Age | 1.00 | 0.99 |
| Sex-Male | 1.29 | 0.80 |
| Race |  |  |
| Black/African American | 0.75 | 0.84 |
| White | 0.43 | 0.32 |
| ECOG PS |  |  |
| 1 | 1.88 | 0.46 |
| 2 | NA^c^ | NA^c^ |
| CNS metastases at baseline | 9.00 | 0.02 |
| Histology-Adenocarcinoma to Others^d^ | NA^e^ | NA^e^ |
| Pharos outcome | 1.88 | 0.49 |
| Clinical response (CR/PR) | 0.98 | 0.97 |

^a^Samples with missing covariate as an independent factor were removed from the corresponding logistic regression model ^b^All combined non-adenocarcinoma histologies, which included pleomorphic, squamous cell, sarcomatoid, neuroendocrine, spindle cell, histological and unclassified/undifferentiated carcinomas ^c^All patients with ECOG PS of 2 had positive results per F1L CDx ^d^All combined non-adenocarcinoma histologies, which included: carcinomas with pleomorphic, sarcomatoid or sarcomatous elements, cytological, histological, neuroendocrine, spindle cell, squamous cell carcinoma, unclassified/undifferentiated carcinoma or ‘other’ ^e^All patients with non-adenocarcinoma histologies had positive results per F1L CDx
CNS, central nervous system; CR, complete response; ECOG PS, Eastern Cooperative Oncology Group performance status; F1L CDx, FoundationOne Liquid CDx; fp, fusion-positive; NA, not applicable; NSCLC, non-small cell lung cancer; NTRK, neurotrophic tyrosine receptor kinase; PR, partial response; ROS1, ROS proto-oncogene 1.

**Table S10** Sensitivity of PPA and NPA according to baseline characteristics, Pharos result and response^a^

|  | **Model 1^b^**  **Median (95% CI)** | **Model 2^c^**  **Median (95% CI)** | **Model 3^d^**  **Median (95% CI)** |
| --- | --- | --- | --- |
| ***NTRK* gene fusion** | | | |
| PPA | 46.3 (40.7–51.9) | 46.3 (37.0–51.4) | 46.7 (40.7–55.1) |
| NPA^e^ | 100 (100–100) | 100 (100–100) | 100 (100–100) |
| ***ROS1* gene fusion** | | | |
| PPA | 55.8 (51.0–65.8) | 60.8 (51.4–68.2) | 62.7 (52.9–69.6) |
| NPA^e^ | 100 (100–100) | 100 (100–100) | 100 (100–100) |

^a^Imputation for missing F1L CDx results (*NTRK-*fp samples, *n* = 11; *ROS1-*fp samples, *n* = 22), using multiple imputation with 50 imputed data sets, applied for three imputation models; Independent factors used in each model: ^b^age, sex, race, ECOG PS, CNS lesion, histology, Pharos result, ^c^treatment response, Pharos result, ^d^treatment response; ^e^There was no variability in NPA for the imputation datasets
CI, confidence interval; CNS, central nervous system; ECOG PS, Eastern Cooperative Oncology Group performance score; F1L CDx, FoundationOne Liquid CDx; fp, fusion-positive; NPA, negative percent agreement; PPA, positive percent agreement; NTRK, neurotrophic tyrosine receptor kinase; ROS1, ROS proto-oncogene 1.

**Table S11** Sensitivity of PPA and NPA according to CTA used^a^

|  | **Pharos**  **Median (95% CI)** | **F1/F1Heme**  **Median (95% CI)** | **Others**  **Median (95% CI)** |
| --- | --- | --- | --- |
| ***NTRK* gene fusion  (*N* = 54)** | (*n* = 21) | (*n* = 19) | (*n* = 14) |
| PPA | 47.6 (38.1–52.3) | 57.1 (42.7–64.2) | 31.6 (22.3–46.2) |
| NPA^b^ | 100 (100–100) | 100 (100–100) | 100 (100–100) |
| ***ROS1* gene fusion  (*N* = 51)** | (*n* = 23) | (*n* = 5) | (*n* = 23) |
| PPA | 60.8 (52.2–65.2) | 100 (100–100) | 60.8 (47.8–69.6) |
| NPA^b^ | 100 (100–100) | 100 (100–100) | 100 (100–100) |

^a^Imputation for unevaluable F1L CDx results (*NTRK-*fp samples, *n* = 11; *ROS1-*fp samples, *n* = 22), using Model 3 with treatment response as an independent factor, PPA and NPA were calculated for each subset based on 50 imputation datasets
^b^There was no variability in NPA for the imputation datasets
CI, confidence interval; CTA, clinical trial assay; F1L CDx, FoundationOne Liquid CDx; fp, fusion-positive; NPA, negative percent agreement; PPA, positive percent agreement; NTRK, neurotrophic tyrosine receptor kinase; ROS1, ROS proto-oncogene 1.

**Table S12** Robustness of ORR using imputation Model 1 for *NTRK*-fp samples

|  | **Median imputed ORR, % (IQR)** | | **Median observed ORR, % (95% CI)** | |
| --- | --- | --- | --- | --- |
|  | **CTA+ F1LCDx+** | **CTA+ F1LCDx-** | **CTA+ F1LCDx+** | **CTA+ F1LCDx-** |
| *NTRK*-fp samples | 64 (63–67)  *n* = 29 | 52 (50–55)  *n* = 22 | 72.2 (46.5–90.3)  *n* = 18 | 55.0 (31.5–76.9)  *n* = 20 |

CI, confidence intervals; CTA, clinical trial assay; F1L CDx, FoundationOne Liquid CDx; fp, fusion-positive; IQR, interquartile range; NTRK, neurotrophic tyrosine receptor kinase; ORR, objective response rate.

**Table S13** Robustness of ORR using imputation Model 1 for *ROS1­-­*fp samples

|  | **Median imputed ORR, % (IQR)** | | **Median observed ORR, % (95% CI)** | |  |
| --- | --- | --- | --- | --- | --- |
|  | **CTA+ F1LCDx+** | **CTA+ F1LCDx-** | **CTA+ F1LCDx+** | **CTA+ F1LCDx-** | |
| *ROS1*-fp samples | 78 (76–79)  *n* = 29 | 79 (77–81)  *n* = 22 | 72.2 (46.5–90.3)  *n* = 18 | 72.7 (39.0–94.0)  *n* = 11 | |

CI, confidence intervals; CTA, clinical trial assay; F1L CDx, FoundationOne Liquid CDx; fp, fusion-positive; IQR, interquartile range; ORR, objective response rate; ROS1, ROS proto-oncogene 1.

**SUPPLEMENTARY FIGURES**

**Fig. S1** Differences in sum of the longest diameters between tumors where *ROS1* or *NTRK* fusions were detected by F1L CDx versus those with no detected fusion

A) *NTRK1/3* fusions; B) *ROS1* fusions


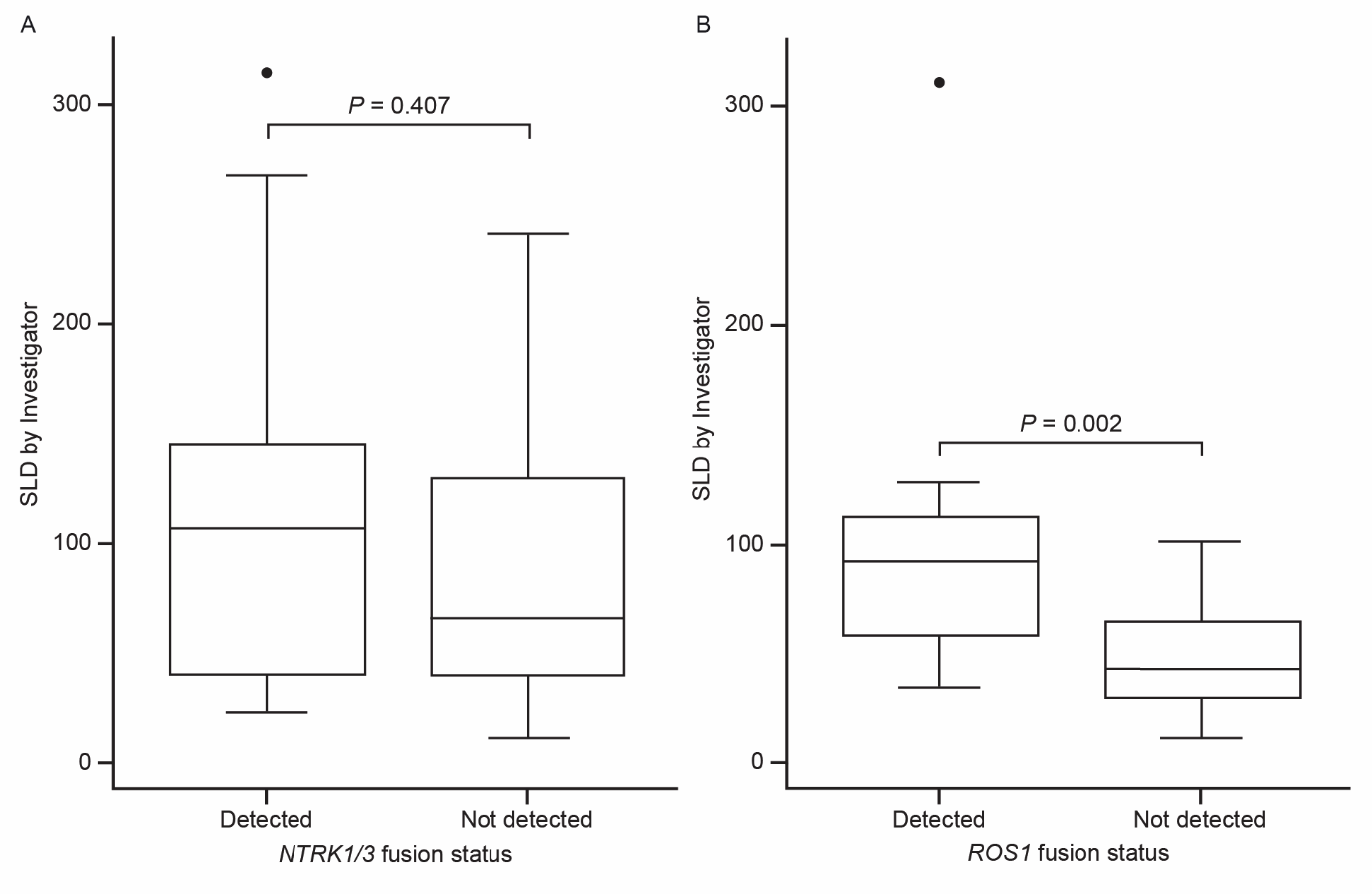


F1L CDx, FoundationOne Liquid biopsy CDx; fp, fusion positive; NTRK, neurotrophic tyrosine receptor kinase; ROS1, ROS proto-oncogene 1; SLD, sum of the longest diameters.

**Fig. S2** Duration of response in patients with *NTRK* fusion-positive solid tumors and *ROS1* fusion-positive NSCLC by ctDNA fusion status

A) *NTRK1/3* fusions; B) *ROS1* fusions

**
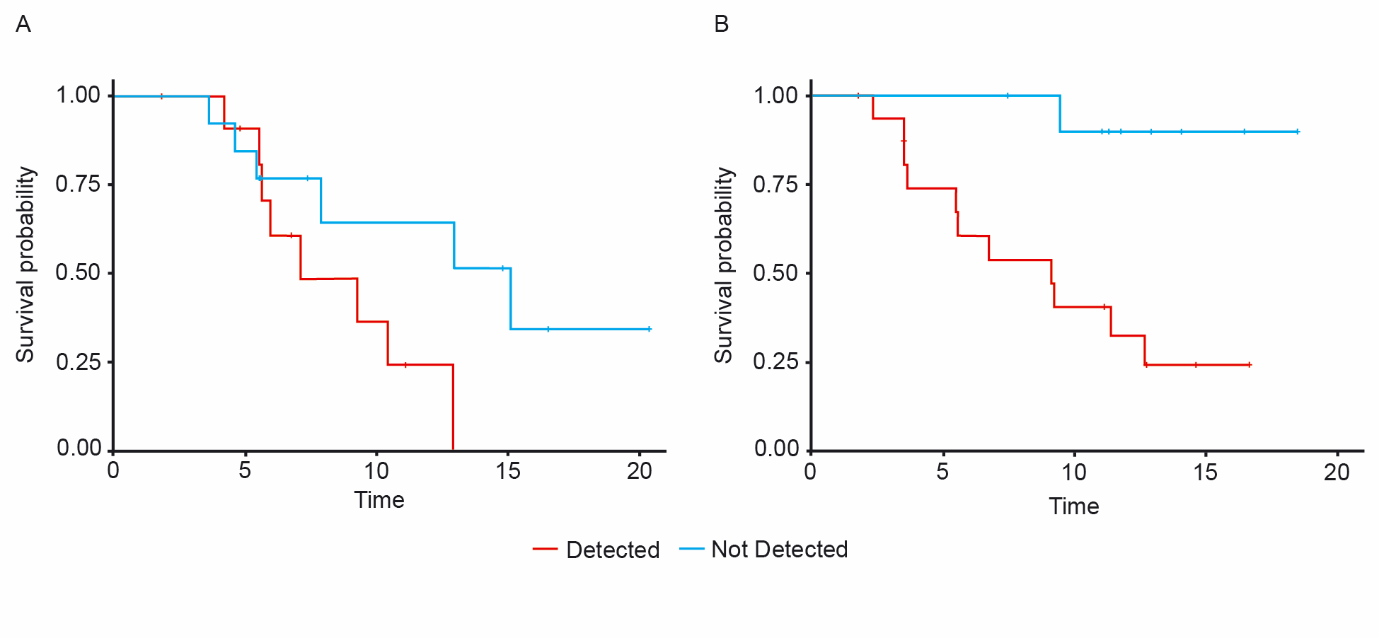
**

F1L CDx, FoundationOne Liquid biopsy CDx; fp, fusion positive; NTRK, neurotrophic tyrosine receptor kinase; ROS1, ROS proto-oncogene 1; SLD, sum of the longest diameters.

**Fig. S3** F1L CDx detection of primary and secondary mutations in *NTRK1/2/3* and *ROS1* fusion-positive samples

A) Primary *NTRK1/2/3* fusion; B) Primary *ROS1* fusion; C) Secondary *NTRK1/2/3* mutation; D) Secondary *ROS1* mutation


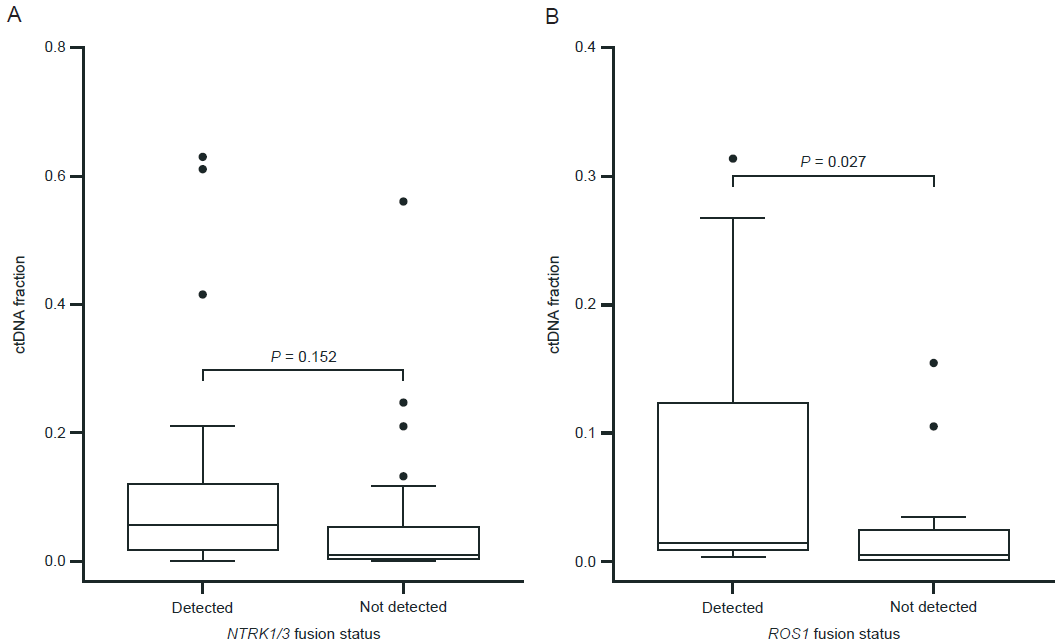

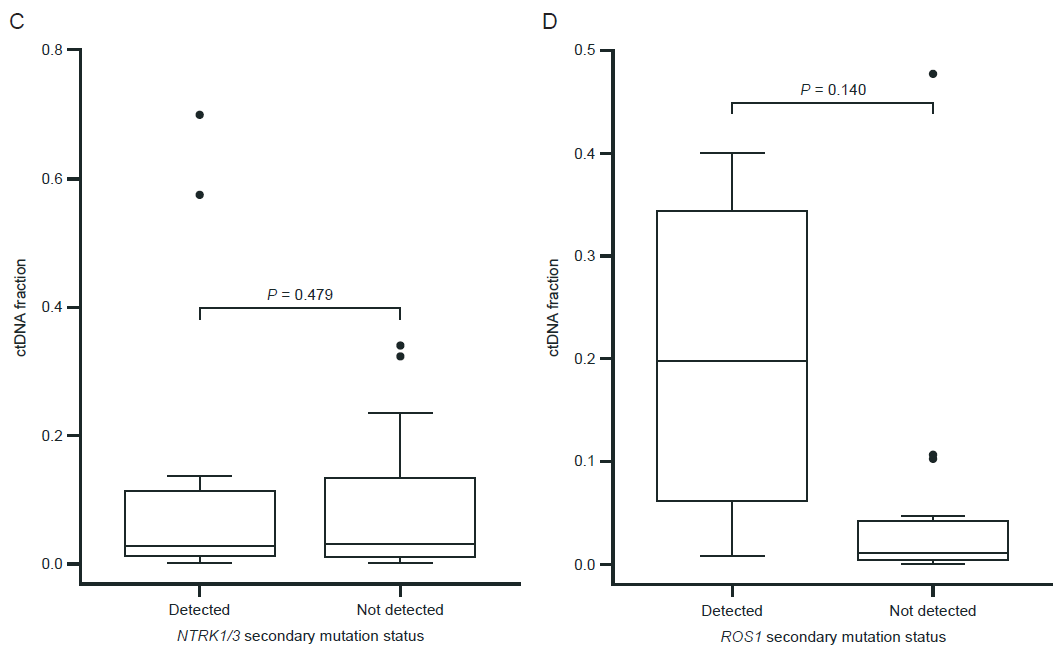


F1L CDx, FoundationOne Liquid biopsy CDx; NTRK, neurotrophic tyrosine receptor kinase; ROS1, ROS proto-oncogene 1.

**References**

1. Li M (2015) Statistical consideration and challenges in bridging study of personalized medicine. *J Biopharm Stat* **25**, 397–407.
